# Supplementary material for: Contributions of neighborhood social environment and air pollution exposure to Black-White disparities in epigenetic aging
Source: PLoS One. 2023 Jul 5;18(7):e0287112. doi: 10.1371/journal.pone.0287112 (PMC10321643; doi:10.1371/journal.pone.0287112)
Supplement: S1 Table — Mean DNAm aging is shown in unscaled units. (PDF) [file pone.0287112.s001.pdf]

S1 Table. Racial disparity in DNAm aging with weights or ancestry-informative principal components.

Characteristic	White, N = 2,438[1]	Black, N = 522[1]	Difference[2]	p-value[2]	Effect Size[3]
GrimAge raw residual	-0.34 (4.68)	1.09 (4.86)	-1.4	<0.001	0.299
GrimAge weighted residual	-0.12 (4.68)	1.33 (4.86)	-1.4	<0.001	0.304
GrimAge residual with ancestry-informative PCs	-0.34 (4.64)	1.05 (4.82)	-1.4	<0.001	0.295
Unknown	265	98			
DPoAm raw residual	-0.01 (0.09)	0.02 (0.10)	-0.03	<0.001	0.324
DPoAm weighted residual	0.00 (0.09)	0.03 (0.10)	-0.03	<0.001	0.334
DPoAm residual with ancestry-informative PCs	-0.01 (0.09)	0.02 (0.09)	-0.03	<0.001	0.336
Unknown	265	98			

Mean DNAm aging is shown in unscaled units.

[1]Mean (SD)

[2]Welch Two Sample t-test

[3]Cohen's D
