# Supplementary material for: Contributions of neighborhood social environment and air pollution exposure to Black-White disparities in epigenetic aging
Source: PLoS One. 2023 Jul 5;18(7):e0287112. doi: 10.1371/journal.pone.0287112 (PMC10321643; doi:10.1371/journal.pone.0287112)
Supplement: S2 Table — Results of linear regression models with GrimAge aging as the outcome excluding 667 participants whose residential census tract changed. (PDF) [file pone.0287112.s002.pdf]

S2 Table. GrimAge aging: Multivariable regression models excluding individuals who moved 2010-2016.

GrimAge[1]	Total disparity[1]	Individual SES[1]	SDI[1]	Social Disorder[1]	Physical Disorder[1]	PM2.5[1]	Ozone[1]	NO_2[1]
**Race**								
White	—	—	—	—	—	—	—	—
Black	0.24**	0.07	0.02	0.06	0.06	0.06	0.06	0.06
	(0.10,0.37)	(-0.06,0.19)	(-0.11,0.15)	(-0.07,0.18)	(-0.07,0.19)	(-0.07,0.18)	(-0.07,0.19)	(-0.07,0.18)
**Gender**								
Male		—	—	—	—	—	—	—
Female		-0.70***	-0.69***	-0.69***	-0.70***	-0.70***	-0.70***	-0.70***
		(-0.77,-0.62)	(-0.77,-0.62)	(-0.76,-0.62)	(-0.77,-0.62)	(-0.77,-0.62)	(-0.77,-0.62)	(-0.77,-0.62)
**Education**								
College +		—	—	—	—	—	—	—
Some College		0.16**	0.15*	0.15**	0.16**	0.15**	0.16**	0.16**
		(0.06,0.25)	(0.06,0.25)	(0.06,0.25)	(0.06,0.25)	(0.06,0.25)	(0.06,0.25)	(0.06,0.25)
High School		0.22***	0.21***	0.22***	0.22***	0.21***	0.22***	0.22***
		(0.12,0.31)	(0.12,0.31)	(0.12,0.31)	(0.12,0.31)	(0.12,0.31)	(0.12,0.31)	(0.12,0.31)
< High School		0.42***	0.42***	0.42***	0.42***	0.42***	0.42***	0.43***
		(0.28,0.56)	(0.28,0.56)	(0.28,0.56)	(0.28,0.56)	(0.28,0.56)	(0.28,0.56)	(0.28,0.57)
**Quartile Wealth/Income**								
4		—	—	—	—	—	—	—
3		0.19***	0.18**	0.19***	0.19***	0.19***	0.19***	0.20***
		(0.10,0.29)	(0.09,0.28)	(0.09,0.28)	(0.10,0.29)	(0.10,0.29)	(0.10,0.29)	(0.10,0.29)
2		0.44***	0.41***	0.43***	0.44***	0.44***	0.44***	0.44***
		(0.33,0.54)	(0.30,0.52)	(0.33,0.54)	(0.33,0.54)	(0.34,0.54)	(0.33,0.54)	(0.34,0.55)
1		0.54***	0.50***	0.53***	0.53***	0.54***	0.54***	0.54***
		(0.42,0.66)	(0.38,0.63)	(0.41,0.65)	(0.41,0.66)	(0.42,0.66)	(0.42,0.66)	(0.42,0.66)
**Neighborhood Exposure**			0.05	0.02	0.01	0.01	0.00	0.00
			(0.01,0.09)	(-0.02,0.06)	(-0.03,0.05)	(-0.01,0.03)	(-0.01,0.01)	(0.00,0.01)
**(Intercept)**	-0.06*	-0.07	-0.03	-0.07	-0.07	-0.17	0.07	-0.11
	(-0.11,-0.02)	(-0.15,0.01)	(-0.12,0.05)	(-0.15,0.02)	(-0.15,0.02)	(-0.36,0.02)	(-0.29,0.43)	(-0.22,0.01)
R^2	0.005	0.198	0.200	0.198	0.198	0.199	0.198	0.198
AIC	6,842	6,362	6,359	6,363	6,363	6,362	6,363	6,363
No. Obs.	2,292	2,292	2,292	2,292	2,292	2,292	2,292	2,292

Results of linear regression models with GrimAge aging as the outcome excluding 667 participants whose residential census tract changed.

[1]ß (95% confidence interval) *p<0.05; **p<0.01; ***p<0.001
