# Supplementary material for: Contributions of neighborhood social environment and air pollution exposure to Black-White disparities in epigenetic aging
Source: PLoS One. 2023 Jul 5;18(7):e0287112. doi: 10.1371/journal.pone.0287112 (PMC10321643; doi:10.1371/journal.pone.0287112)
Supplement: S4 Table — Results of linear regression models with DPoAm aging as the outcome. (PDF) [file pone.0287112.s004.pdf]

S4 Table: DPoAm aging: Interactions between neighborhood exposures and race

**DPoAm[1]**	**SDI[1]**	**Social Disorder[1]**	**Physical Disorder[1]**	**PM2.5 (2014)[1]**	**Ozone (2014)[1]**	**NO_2 (2010)[1]**	**PM2.5 (2010)[1]**
**Race**							
White	—	—	—	—	—	—	—
Black	0.15	0.17	0.16	-0.81	-0.56	-0.08	-0.02
	(0.00,0.31)	(0.04,0.30)	(0.03,0.29)	(-1.5,-0.09)	(-1.9,0.74)	(-0.35,0.19)	(-0.78,0.73)
**Gender**							
Male	—	—	—	—	—	—	—
Female	-0.20***	-0.20***	-0.20***	-0.20***	-0.20***	-0.20***	-0.20***
	(-0.27,-0.13)	(-0.27,-0.12)	(-0.27,-0.13)	(-0.27,-0.13)	(-0.27,-0.13)	(-0.27,-0.13)	(-0.27,-0.13)
**Education**							
College +	—	—	—	—	—	—	—
Some College	0.17**	0.18**	0.18**	0.17**	0.18***	0.18***	0.18**
	(0.08,0.27)	(0.08,0.27)	(0.08,0.27)	(0.08,0.27)	(0.09,0.28)	(0.09,0.27)	(0.08,0.27)
High School	0.22***	0.23***	0.22***	0.23***	0.23***	0.23***	0.22***
	(0.13,0.32)	(0.13,0.32)	(0.13,0.32)	(0.13,0.32)	(0.13,0.32)	(0.13,0.32)	(0.13,0.32)
< High School	0.39***	0.39***	0.39***	0.39***	0.40***	0.39***	0.39***
	(0.25,0.52)	(0.25,0.53)	(0.25,0.53)	(0.26,0.53)	(0.26,0.53)	(0.26,0.53)	(0.26,0.53)
**Quartile Wealth/Income**							
4	—	—	—	—	—	—	—
3	0.05	0.06	0.06	0.06	0.06	0.06	0.06
	(-0.04,0.15)	(-0.04,0.15)	(-0.03,0.16)	(-0.03,0.16)	(-0.04,0.15)	(-0.04,0.15)	(-0.04,0.16)
2	0.18**	0.19**	0.20**	0.20***	0.19**	0.20**	0.19**
	(0.07,0.28)	(0.09,0.29)	(0.09,0.30)	(0.09,0.30)	(0.09,0.30)	(0.09,0.30)	(0.09,0.30)
1	0.29***	0.31***	0.32***	0.32***	0.32***	0.32***	0.32***
	(0.18,0.41)	(0.19,0.42)	(0.20,0.43)	(0.20,0.43)	(0.21,0.44)	(0.21,0.43)	(0.21,0.44)
**Neighborhood Exposure**	0.04	0.02	0.00	0.00	0.00	0.00	0.00
	(-0.01,0.08)	(-0.02,0.06)	(-0.04,0.04)	(-0.02,0.01)	(-0.01,0.00)	(-0.01,0.01)	(-0.02,0.02)
**Race * Neighborhood Exposure**							
Black * Neighborhood Exposure	0.04	0.07	0.11	0.10*	0.02	0.03	0.02
	(-0.10,0.17)	(-0.05,0.19)	(-0.01,0.23)	(0.03,0.17)	(-0.01,0.05)	(0.00,0.05)	(-0.05,0.09)
**(Intercept)**	-0.20***	-0.22***	-0.22***	-0.19	-0.07	-0.22**	-0.26
	(-0.28,-0.11)	(-0.30,-0.14)	(-0.31,-0.14)	(-0.38,0.00)	(-0.42,0.28)	(-0.34,-0.10)	(-0.47,-0.06)
R^2	0.060	0.060	0.060	0.061	0.059	0.061	0.059
AIC	8,843	8,843	8,843	8,839	8,845	8,841	8,807

Results of linear regression models with DPoAm aging as the outcome.
[1]ß (95% confidence interval) *p<0.05; **p<0.01; ***p<0.001
