# Supplementary material for: Contributions of neighborhood social environment and air pollution exposure to Black-White disparities in epigenetic aging
Source: PLoS One. 2023 Jul 5;18(7):e0287112. doi: 10.1371/journal.pone.0287112 (PMC10321643; doi:10.1371/journal.pone.0287112)
Supplement: S5 Table — Results of linear regression models with GrimAge aging as the outcome. (PDF) [file pone.0287112.s005.pdf]

S5 Table: GrimAge aging: Interactions between neighborhood exposures and race

GrimAge[1]	SDI[1]	Social Disorder[1]	Physical Disorder[1]	PM2.5 (2014)[1]	Ozone (2014)[1]	NO_2 (2010)[1]	PM2.5 (2010)[1]
**Race**							
White	—	—	—	—	—	—	—
Black	0.15	0.10	0.13	0.06	-0.81	0.20	-0.02
	(0.01,0.29)	(-0.02,0.22)	(0.01,0.25)	(-0.61,0.73)	(-2.0,0.39)	(-0.05,0.45)	(-0.78,0.73)
**Gender**							
Male	—	—	—	—	—	—	—
Female	-0.73***	-0.72***	-0.73***	-0.73***	-0.73***	-0.73***	-0.20***
	(-0.79,-0.66)	(-0.79,-0.66)	(-0.79,-0.66)	(-0.80,-0.67)	(-0.80,-0.67)	(-0.80,-0.67)	(-0.27,-0.13)
**Education**							
College +	—	—	—	—	—	—	—
Some College	0.25***	0.25***	0.25***	0.26***	0.26***	0.26***	0.18**
	(0.17,0.34)	(0.17,0.34)	(0.17,0.34)	(0.17,0.34)	(0.17,0.35)	(0.17,0.34)	(0.08,0.27)
High School	0.29***	0.30***	0.29***	0.29***	0.30***	0.30***	0.22***
	(0.21,0.38)	(0.21,0.38)	(0.21,0.38)	(0.21,0.38)	(0.21,0.38)	(0.21,0.38)	(0.13,0.32)
< High School	0.50***	0.50***	0.50***	0.51***	0.51***	0.51***	0.39***
	(0.38,0.63)	(0.38,0.63)	(0.38,0.63)	(0.38,0.63)	(0.38,0.64)	(0.38,0.63)	(0.26,0.53)
**Quartile Wealth/Income**							
4	—	—	—	—	—	—	—
3	0.14*	0.15**	0.15**	0.15**	0.15**	0.15**	0.06
	(0.05,0.22)	(0.06,0.23)	(0.06,0.24)	(0.06,0.24)	(0.06,0.24)	(0.06,0.24)	(-0.04,0.16)
2	0.37***	0.40***	0.40***	0.41***	0.41***	0.41***	0.19**
	(0.27,0.47)	(0.30,0.49)	(0.31,0.50)	(0.31,0.50)	(0.31,0.50)	(0.31,0.50)	(0.09,0.30)
1	0.49***	0.52***	0.52***	0.53***	0.53***	0.54***	0.32***
	(0.38,0.60)	(0.41,0.62)	(0.42,0.63)	(0.43,0.64)	(0.43,0.64)	(0.43,0.64)	(0.21,0.44)
**Neighborhood Exposure**	0.07**	0.04	0.02	0.00	0.00	0.00	0.00
	(0.03,0.11)	(0.00,0.07)	(-0.02,0.06)	(-0.02,0.02)	(-0.01,0.01)	(-0.01,0.01)	(-0.02,0.02)
**Race * Neighborhood Exposure**							
Black * Neighborhood Exposure	-0.11	0.03	-0.02	0.01	0.03	-0.01	0.02
	(-0.24,0.01)	(-0.08,0.14)	(-0.13,0.09)	(-0.06,0.07)	(-0.01,0.06)	(-0.03,0.01)	(-0.05,0.09)
**(Intercept)**	-0.05	-0.08	-0.08	-0.11	-0.08	-0.11	-0.26
	(-0.13,0.03)	(-0.16,-0.01)	(-0.16,-0.01)	(-0.29,0.06)	(-0.41,0.25)	(-0.22,0.00)	(-0.47,-0.06)
R^2	0.216	0.214	0.213	0.213	0.214	0.213	0.059
AIC	8,385	8,392	8,395	8,397	8,394	8,396	8,807

Results of linear regression models with GrimAge aging as the outcome.

[1]ß (95% confidence interval) *p<0.05; **p<0.01; ***p<0.001
