# Supplementary material for: Contributions of neighborhood social environment and air pollution exposure to Black-White disparities in epigenetic aging
Source: PLoS One. 2023 Jul 5;18(7):e0287112. doi: 10.1371/journal.pone.0287112 (PMC10321643; doi:10.1371/journal.pone.0287112)
Supplement: S6 Table — Results of linear regression models with DPoAm aging as the outcome. (PDF) [file pone.0287112.s006.pdf]

S6 Table. DPoAm aging: Interactions between neighborhood and social determinants and PM2.5 pollution exposure.

DPoAm[1]	Race[1]	Gender[1]	Individual SES[1]	Education[1]	SDI[1]	Social Disorder[1]	Physical Disorder[1]
**Race**							
White	—	—	—	—	—	—	—
Black	-0.81 (-1.5,-0.09)	0.21** (0.09,0.33)	0.19** (0.07,0.32)	0.20** (0.08,0.32)	0.16 (0.03,0.29)	0.19* (0.07,0.31)	0.20** (0.08,0.32)
**PM2.5**	0.00 (-0.02,0.01)	0.03 (0.01,0.06)	-0.03 (-0.06,0.00)	-0.01 (-0.04,0.02)	0.01 (-0.01,0.03)	0.00 (-0.01,0.02)	0.00 (-0.01,0.02)
**Gender**							
Male	—	—	—	—	—	—	—
Female	-0.20*** (-0.27,-0.13)	0.30 (-0.05,0.65)	-0.20*** (-0.27,-0.13)	-0.20*** (-0.27,-0.13)	-0.20*** (-0.27,-0.13)	-0.19*** (-0.26,-0.12)	-0.20*** (-0.27,-0.13)
**Education**							
College +	—	—	—	—	—	—	—
Some College	0.17** (0.08,0.27)	0.18*** (0.09,0.27)	0.18** (0.08,0.27)	0.02 (-0.41,0.45)	0.17** (0.08,0.27)	0.18** (0.09,0.27)	0.18** (0.08,0.27)
High School	0.23*** (0.13,0.32)	0.23*** (0.13,0.32)	0.22*** (0.13,0.31)	0.20 (-0.24,0.63)	0.22*** (0.13,0.32)	0.23*** (0.14,0.32)	0.23*** (0.13,0.32)
< High School	0.39*** (0.26,0.53)	0.40*** (0.26,0.53)	0.38*** (0.24,0.52)	-0.33 (-1.1,0.40)	0.38*** (0.24,0.52)	0.39*** (0.25,0.52)	0.39*** (0.25,0.53)
**Quartile Wealth/Income**							
4	—	—	—	—	—	—	—
3	0.06 (-0.03,0.16)	0.06 (-0.04,0.15)	-0.52 (-1.0,-0.07)	0.06 (-0.04,0.15)	0.06 (-0.04,0.15)	0.06 (-0.04,0.15)	0.06 (-0.04,0.16)
2	0.20*** (0.09,0.30)	0.20** (0.09,0.30)	-0.07 (-0.55,0.41)	0.20** (0.09,0.30)	0.18** (0.08,0.29)	0.19** (0.09,0.29)	0.19** (0.09,0.30)
1	0.32*** (0.20,0.43)	0.33*** (0.21,0.44)	-0.30 (-0.79,0.19)	0.32*** (0.20,0.43)	0.30*** (0.18,0.42)	0.31*** (0.19,0.42)	0.32*** (0.20,0.43)
**Race * PM2.5**							
Black * PM2.5	0.10* (0.03,0.17)						
**Gender * PM2.5**							
Female * PM2.5		-0.05* (-0.09,-0.02)					
**Quartile Wealth/Income * PM2.5**							
3 * PM2.5			0.06 (0.01,0.11)				
2 * PM2.5			0.03 (-0.02,0.08)				
1 * PM2.5			0.07 (0.02,0.12)				
**Education * PM2.5**							
Some College * PM2.5				0.02 (-0.03,0.06)			
High School * PM2.5				0.00 (-0.04,0.05)			
< High School * PM2.5				0.08 (0.00,0.15)			
**Social Deprivation Index**					-0.14 (-0.32,0.05)		

DPoAm[1]	Race[1]	Gender[1]	Individual SES[1]	Education[1]	SDI[1]	Social Disorder[1]	Physical Disorder[1]
**Social Deprivation Index * PM2.5**					0.02 (0.00,0.04)		
**Social Disorder**						-0.10 (-0.28,0.09)	
**Social Disorder * PM2.5**						0.01 (-0.01,0.03)	
**Physical Disorder**							-0.08 (-0.27,0.11)
**Physical Disorder * PM2.5**							0.01 (-0.01,0.03)
**(Intercept)**	-0.19 (-0.38,0.00)	-0.54*** (-0.81,-0.27)	0.08 (-0.22,0.39)	-0.15 (-0.45,0.15)	-0.27* (-0.46,-0.07)	-0.26* (-0.45,-0.07)	-0.26* (-0.45,-0.07)
R^2	0.061	0.062	0.062	0.060	0.061	0.060	0.059
AIC	8,839	8,838	8,841	8,846	8,842	8,844	8,848

Results of linear regression models with DPoAm aging as the outcome.

[1]ß (95% confidence interval) *p<0.05; **p<0.01; ***p<0.001
