# Supplementary material for: Contributions of neighborhood social environment and air pollution exposure to Black-White disparities in epigenetic aging
Source: PLoS One. 2023 Jul 5;18(7):e0287112. doi: 10.1371/journal.pone.0287112 (PMC10321643; doi:10.1371/journal.pone.0287112)
Supplement: S7 Table — Full results of decomposition for GrimAge and DPoAm. (PDF) [file pone.0287112.s007.pdf]

S7 Table: Threefold decomposition of individual and neighborhood contributions to racial disparity in DNAm aging. Full results of decomposition for GrimAge and DPoAm.

		GrimAge			DPoAm		
Variable	Component	Percent	Estimate	95% CI	Percent	Estimate	95% CI
Overall	Endowments	**58.82**	**0.18**	**(0.11, 0.25)**	**41.57**	**0.14**	**(0.07, 0.21)**
	Coefficients	**67.84**	**0.21**	**(0.05, 0.36)**	30.53	0.10	(-0.05, 0.25)
	Interaction	-26.66	-0.08	(-0.23, 0.07)	27.90	0.09	(-0.05, 0.24)
Intercept	Endowments	0.00	0.00	(0.00, 0.00)	0.00	0.00	(0.00, 0.00)
	Coefficients	-166.61	-0.51	(-1.81, 0.80)	**-407.10**	**-1.37**	**(-2.57, -0.17)**
	Interaction	-0.00	-0.00	(-0.00, 0.00)	-0.00	-0.00	(-0.00, 0.00)
Female	Endowments	**-27.40**	**-0.08**	**(-0.12, -0.05)**	**-6.80**	**-0.02**	**(-0.04, -0.01)**
	Coefficients	30.35	0.09	(-0.02, 0.21)	-13.00	-0.04	(-0.17, 0.08)
	Interaction	5.93	0.02	(-0.01, 0.04)	-2.54	-0.01	(-0.03, 0.02)
Education: Some College	Endowments	0.23	0.00	(-0.00, 0.00)	0.38	0.00	(-0.00, 0.00)
	Coefficients	13.44	0.04	(-0.00, 0.09)	**22.63**	**0.08**	**(0.03, 0.12)**
	Interaction	-0.87	-0.00	(-0.01, 0.01)	-1.46	-0.00	(-0.02, 0.01)
High School	Endowments	-0.01	-0.00	(-0.00, 0.00)	-0.03	-0.00	(-0.00, 0.00)
	Coefficients	10.98	0.03	(-0.02, 0.08)	1.66	0.01	(-0.05, 0.06)
	Interaction	-0.06	-0.00	(-0.01, 0.01)	-0.01	-0.00	(-0.00, 0.00)
Less than High School	Endowments	**11.10**	**0.03**	**(0.02, 0.05)**	**6.73**	**0.02**	**(0.01, 0.04)**
	Coefficients	-7.84	-0.02	(-0.04, -0.00)	-4.80	-0.02	(-0.04, 0.00)
	Interaction	-10.10	-0.03	(-0.06, -0.00)	-6.19	-0.02	(-0.05, 0.01)
Wealth/Income: 3rd Quartile	Endowments	2.02	0.01	(-0.00, 0.01)	1.52	0.01	(-0.00, 0.01)
	Coefficients	-13.62	-0.04	(-0.09, 0.01)	-11.83	-0.04	(-0.10, 0.02)
	Interaction	6.03	0.02	(-0.01, 0.04)	5.24	0.02	(-0.01, 0.04)
2nd Quartile	Endowments	1.01	0.00	(-0.00, 0.01)	0.13	0.00	(-0.00, 0.00)
	Coefficients	1.12	0.00	(-0.04, 0.05)	7.01	0.02	(-0.02, 0.07)
	Interaction	0.17	0.00	(-0.01, 0.01)	1.07	0.00	(-0.00, 0.01)
Lowest Quartile	Endowments	**21.49**	**0.07**	**(0.04, 0.09)**	**14.39**	**0.05**	**(0.02, 0.08)**
	Coefficients	7.37	0.02	(-0.01, 0.06)	5.02	0.02	(-0.02, 0.05)
	Interaction	12.91	0.04	(-0.02, 0.10)	8.79	0.03	(-0.03, 0.09)
Social Deprivation Index	Endowments	**21.09**	**0.06**	**(0.02, 0.11)**	7.02	0.02	(-0.03, 0.08)
	Coefficients	11.73	0.04	(-0.01, 0.08)	3.04	0.01	(-0.03, 0.05)
	Interaction	-40.75	-0.12	(-0.27, 0.02)	-10.57	-0.04	(-0.18, 0.11)
Social Disorder	Endowments	7.26	0.02	(-0.01, 0.05)	7.20	0.02	(-0.01, 0.06)
	Coefficients	1.22	0.00	(-0.02, 0.03)	2.18	0.01	(-0.02, 0.03)
	Interaction	-4.37	-0.01	(-0.09, 0.06)	-7.81	-0.03	(-0.11, 0.06)
Physical Disorder	Endowments	-5.48	-0.02	(-0.05, 0.02)	-4.77	-0.02	(-0.06, 0.02)
	Coefficients	-0.52	-0.00	(-0.03, 0.02)	-4.35	-0.01	(-0.04, 0.01)
	Interaction	1.92	0.01	(-0.08, 0.10)	16.02	0.05	(-0.04, 0.15)
PM2.5	Endowments	-0.09	-0.00	(-0.02, 0.02)	-2.70	-0.01	(-0.03, 0.01)
	Coefficients	-16.62	-0.05	(-0.62, 0.52)	144.90	0.49	(-0.10, 1.07)
	Interaction	-1.60	-0.00	(-0.06, 0.05)	13.95	0.05	(-0.01, 0.10)
Ozone	Endowments	-0.54	-0.00	(-0.01, 0.01)	0.91	0.00	(-0.01, 0.02)
	Coefficients	160.63	0.49	(-0.67, 1.65)	223.56	0.75	(-0.35, 1.86)
	Interaction	-5.46	-0.02	(-0.06, 0.02)	-7.61	-0.03	(-0.06, 0.01)
NO_2	Endowments	1.62	0.00	(-0.02, 0.03)	4.18	0.01	(-0.01, 0.04)
	Coefficients	29.26	0.09	(-0.10, 0.28)	34.05	0.11	(-0.07, 0.30)
	Interaction	9.76	0.03	(-0.03, 0.09)	11.36	0.04	(-0.02, 0.10)
