# Supplementary material for: Contributions of neighborhood social environment and air pollution exposure to Black-White disparities in epigenetic aging
Source: PLoS One. 2023 Jul 5;18(7):e0287112. doi: 10.1371/journal.pone.0287112 (PMC10321643; doi:10.1371/journal.pone.0287112)
Supplement: Attachment — Submitted filename: Response to reviewers 2.docx [file pone.0287112.s009.docx]

**Response to Editor & Reviewers**

Editorial Response

We would like to thank the editors for your consideration of our manuscript and efforts in the review process. We provide point by point responses to all editorial and reviewer comments (italicized below) and highlight all major changes in the body of the manuscript. We feel these minor changes further increase the quality of the manuscript and appreciate the reviewers’ further thoughtful suggestions.

*Journal Requirements: All reviewers responded that all journal requirements have been adequately addressed with one discrepant suggestions about Data Availability. Two reviewers agree that we are in compliance with the PLOS Data policy but one reviewer suggested we were not in compliance. We re-iterate that we clearly outlined data availability in our prior submission which is the required language from the Health & Retirement Study:*

Data used in this study are available through a third-party, the Health and Retirement Study. However, once a researcher has obtained access to the data, they can use our code available at github.com/pennbindlab to recreate the minimal data set. Description of the data set and source: The Health and Retirement Study (HRS) is sponsored by the National Institute on Aging (grant number NIA U01 AG009740) and is conducted by the University of Michigan. It is a longitudinal panel survey study that collects in-depth survey interviews and biological samples from a representative sample of Americans 50 and older. Study participants’ geographic location and Contextual Data Resource data are available only under special agreement because they contain sensitive and/or confidential information.

Verification of permission to use the data set: Restricted Data Agreement # 2021-084 was approved on October 15, 2021. Information to apply to gain access: Researchers must apply for access through HRS at the site https://hrs.isr.umich.edu/data-products/restricted-data. The application and use of data are free of charge. IRB approval is required. All questions related to HRS Restricted data should be sent to hrsrdaapplication@umich.edu.

Reviewers #1 and #3

Thank you for your supportive comments and thoughtful suggestions that we feel by addressing have improved the quality of the manuscript.

Reviewer #2:

We appreciate your attention to detail and have edited the manuscript to change the PM2.5 font and update some suggested references. Specifically:

1. *please subscript the PM2.5, this is a standard format.*

We have changed all instances of “PM2.5” to PM_2.5_. Given this minor change we do not highlight throughout.

*2. I agreed with the response from the authors for Q. 6. However, please cite articles from the past 3 years, because the changes could occur in building characteristics and the association between indoor and outdoor air pollutants.*

We agree with the reviewer that relationships between indoor and outdoor air pollution may change over time. Unfortunately there are technical challenges to accurately measure personal air pollution exposure and data are limited, especially studies that are representative across the United States. A recent review discusses the technological and methodological challenges of measuring personal air pollution exposure ([Brokamp et al. 2019](https://www.sciencedirect.com/science/article/pii/S0091674919306037)). A review and meta-analysis of studies on PM_2.5_ showed that in the United States the correlation between personal and ambient exposure is 0.6 but did not examine NO_2_ nor O_3_ ([Boomhower et al., 2022](https://www.sciencedirect.com/science/article/pii/S0013935121013141)). Another recent review and meta-analysis examined the absolute difference between personal ambient exposure and found greater differences and more variability for NO_2_ than for PM_2.5_ but did not examine O_3_ ([Evangelopoulos et al., 2020](https://www.sciencedirect.com/science/article/pii/S0160412019333719)). We were not able to identify more recent studies comparing personal and ambient exposure of O_3_ in the United States. We added the latter two citations to the manuscript (references 75 and 76) on page 29 (highlighted in yellow in the marked-up version).
